# Enrichment of the Canadian Partnership for Tomorrow’s Health Study: Protocol for Administering Multiple Online Dietary and Movement Behavior Assessment Tools in a Longitudinal Cohort Study

**DOI:** 10.2196/71680

**Published:** 2025-12-02

**Authors:** Rachel A Murphy, Jennifer E Vena, Alyssa D Milano, Guy Faulkner, Benoît Lamarche, Charles E Matthews, Leia M Minaker, Dylan Spicker, Ellen Sweeney, Michael Wallace, Michael J Widener, Sharon I Kirkpatrick

**Affiliations:** 1 School of Population and Public Health Faculty of Medicine University of British Columbia Vancouver, BC Canada; 2 Population Health Sciences BC Cancer Vancouver, BC Canada; 3 Alberta’s Tomorrow Project Alberta Health Services Calgary, AB Canada; 4 School of Kinesiology Faculty of Education University of British Columbia Vancouver, BC Canada; 5 Centre Nutrition, Santé et Société (NUTRISS) School of Nutrition Université Laval Québec, QC Canada; 6 National Cancer Institute Bethesda, MD United States; 7 School of Planning University of Waterloo Waterloo, ON Canada; 8 University of New Brunswick Saint John Saint John, NB Canada; 9 Dalhousie University Halifax, NS Canada; 10 Department of Statistics and Actuarial Science University of Waterloo Waterloo, ON Canada; 11 Department of Geography and Planning University of Toronto Toronto, ON Canada; 12 School of Public Health Sciences University of Waterloo Waterloo, ON Canada

**Keywords:** dietary intake, movement behavior, environments, population cohort, nutrition, web-based assessment

## Abstract

**Background:**

Suboptimal diet quality and physical inactivity are key risk factors for chronic disease and disability in Canada. However, the lack of high-quality population-level data hinders the development of evidence-based strategies to support improvements in diet quality, movement behaviors (physical inactivity, activity, and sleep), and health. The lack of data is also a barrier to developing capacity in diet and physical activity assessment and epidemiology in Canada.

**Objective:**

This protocol describes the development of the largest known repository of dietary intake and movement behavior data in Canada by drawing upon an existing longitudinal cohort study, the Canadian Partnership for Tomorrow’s Health (CanPath). In the short-term, the data will be used to examine associations between system factors (eg, retail food environments) and dietary intake. In the longer-term, data will be available to pursue a range of research questions, including longitudinal associations between diet, movement behavior, and health outcomes.

**Methods:**

Participants in CanPath (>330,000 adults) who can complete online questionnaires are eligible and will be asked to complete a baseline web-based questionnaire including questions on demographic characteristics and screeners capturing dietary intake and movement behaviors. Subsequently, participants will be invited to complete an online 24-hour dietary recall using the Automated Self-Administered 24-Hour Dietary Assessment Tool (ASA24-Canada-2018) and an online 24-hour activity recall using Activities Completed Over Time in 24 Hours (ACT24). Repeat recalls will be administered 1-2 weeks later. A subset of participants will be invited to complete 2 additional ASA24-Canada-2018 and Activities Completed Over Time in 24 Hours recalls 6 months later. One year after baseline, participants will be invited to complete past-year diet and movement behavior questionnaires. In Québec, dietary intake and movement behavior data are from 3000 CanPath participants enrolled in the NutriQuébec study. Participant addresses will be linked to geospatial data on the food, built, and social environment.

**Results:**

Data collection began in 2025. As of manuscript acceptance (November 4, 2025), 3171 participants had been recruited. Data processing and cleaning will be completed in 2027, and analyses will occur in 2028. It is anticipated that dietary intake and movement behavior data will be available for up to 100,000 adults.

**Conclusions:**

This protocol outlines the collection of detailed data on dietary intake and movement behavior in a large cohort spanning all provinces in Canada. In addition to allowing examination of a range of research questions related to diet, movement behavior, and health, the combination of assessment tools will support methodological research, including expanding analytical strategies to mitigate the effects of error in dietary and movement behavior data. This effort will also build capacity in the collection, processing, and harmonization of dietary and movement behavior data among cohorts and provide a training ground for emerging researchers.

**International Registered Report Identifier (IRRID):**

PRR1-10.2196/71680

## Introduction

Consumption of high-quality diets rich in fruits and vegetables, whole grains, dairy, and plant-based foods and low in refined grains, added sugars, and sodium is essential for overall health and chronic disease prevention [1-3]. Most adults in Canada consume diets that do not meet dietary guidelines [4-7], with little evidence of improvement over time [5,8]. Accordingly, poor diet quality is the leading cause of death and disability in Canada [9]. Healthy movement behaviors—including engaging in physical activity, muscle strengthening, and standing; limiting sedentary time; and getting good quality sleep—are also critical for health and chronic disease prevention [10,11]. Most adults in Canada do not meet guidelines for movement behavior, with variation according to sex, age, family arrangement, and health status [12].

As part of the first-ever Food Policy for Canada, the federal government announced approximately US $35.4 million investment in local infrastructure to improve access to healthy foods in communities [13]. However, large gaps in population-level evidence on the retail food environment (RFE) and overall dietary intake make it unclear how to achieve the desired outcome of improving diet quality. Evidence has shown that improving access to healthy foods, for example, by introducing new grocery stores or stores selling a wide variety of healthy food in a neighborhood, has a null or modest impact on dietary intake [14-16]. Proximity to fast food outlets is linked to (poor) diet quality, although not consistently [16]. Methodological limitations within studies, including lack of standardized measures of the RFE, sparse dietary intake data (typically confined to fruit and vegetable consumption), and absence of consideration of individual-level factors that interact with dietary intake (eg, sex, gender, income, family structure, education, and sedentary behavior), hinder the ability to draw firm conclusions [15]. Research and policy efforts to improve the RFE seldom consider the multidimensionality of factors within which eating patterns emerge and the complexity of neighborhood environments where built and social environment and individual-level factors interact with the RFE to affect dietary intake. For example, a new grocery store may have a limited impact on food consumption in low-income neighborhoods unless there is adequate walkability (eg, sidewalks), public transit access, and social support, and individuals have adequate income to purchase food at the new store. Similarly, the federal government developed “A Common Vision for Increasing Physical Activity and Reducing Sedentary Living in Canada: Let’s Move” [17]. A Common Vision recognizes the complex system of factors that contribute to movement behavior, including neighborhood-level factors such as social environments and physical environments.

To inform interventions to improve diet quality, physical activity, and health, we need high-quality comprehensive data on dietary intake and movement behaviors that reflect individuals in Canada and the diverse environments in which they live. Existing population-level surveys such as the Canadian Community Health Survey and Canadian Health Measures Survey [18,19] do not provide contemporary estimates of dietary intake and overall diet quality. Comprehensive dietary data have only been collected (using a single 24-hour recall for most participants) twice in recent decades in the Canadian Community Health Survey [20,21], with the most recent data collected in 2015. The annual component of the Canadian Community Health Survey is limited to querying fruit and vegetable intake. In the Canadian Health Measures Survey, dietary data are limited to intake of select food groups [22]. Movement behavior is surveyed as part of the annual component of the Canadian Community Health Survey, providing data at the health region level every 2 years [23]. The lack of concurrent measurement of dietary intake and movement behavior in the Canadian Community Health Survey and the limited ability to link to information on health outcomes constrain the use of these existing data to inform interventions. Established population health cohorts are ideal for the collection of high-quality dietary intake and movement behavior data, given the ability to leverage existing infrastructure. The longitudinal follow-up of participants and the ability to link with administrative health data, including on health outcomes, within cohorts allow the use of the dietary and movement behavior data to address a broad range of research questions.

This protocol describes the enrichment of the Canadian Partnership for Tomorrow’s Health (CanPath) [24] to create the largest known repository of dietary intake and movement behavior data in Canada. In the short-term, the newly collected dietary intake data will be used to examine associations between built, social, and RFE and dietary intake as part of the Healthy Eating and Supportive Environments (HEAL) study. Specifically, HEAL aims (1) to evaluate associations between the RFE and diet quality as measured by the Healthy Eating Food Index-2019 and (2) to evaluate whether associations between the RFE and diet quality vary by environmental factors (such as built and social environmental characteristics) and individual-level factors (such as gender identity and income). Beyond the HEAL-specific aims, this rich resource of data will enable addressing timely research questions on dietary intake, including how it is associated with food security, discrimination, and financial stress. In the longer term, the dietary intake and movement behavior data collected as part of HEAL—in combination with the wealth of information already held by CanPath—will make it possible to pursue a range of questions including how dietary and movement patterns are associated with health outcomes such as mortality and incident chronic disease. The combination of assessment tools administered in HEAL will support methodological research, including expanding strategies to mitigate the effects of error in dietary intake and movement behavior data on observed associations with health. Finally, this effort will build capacity in the collection, processing, and harmonization of dietary intake and movement behavior data among cohorts and provide a training ground for emerging researchers.

## Methods

### Data Sources

#### Overview

**Figure 1:**
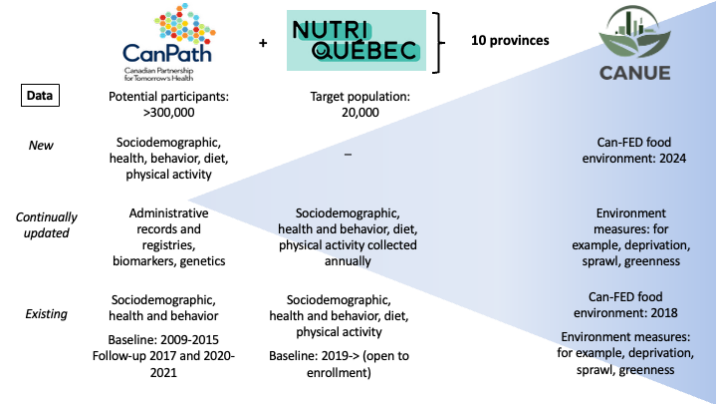
Overview of the data sources comprising the Healthy Eating and Supportive Environments study [23-26]. Can-FED: Canadian Food Environment Dataset; CanPath: Canadian Partnership for Tomorrow’s Health; CANUE: Canadian Urban Environmental Health Research Consortium.

HEAL is leveraging 4 national resources: CanPath [24], the NutriQuébec study [25], the Canadian Urban Environmental Health Research Consortium (CANUE) [26], and the Canadian Food Environment Dataset (Can-FED) [27] (Figure 1).

#### Canadian Partnership for Tomorrow’s Health

CanPath is one of the world’s largest prospective health research platforms, bringing together data from 7 regional cohorts including over 330,000 adults living in 10 provinces [24]. The CanPath regional cohorts include Alberta’s Tomorrow Project (n=~55,000, recruited from 2000 to 2015) [28,29], the BC Generations Project (n=~30,000, recruited from 2009 to 2016) [30,31], the Atlantic Partnership for Tomorrow’s Health (n=~35,000, recruited from 2009 to 2015) [32,33], the Ontario Health Study (n=~225,000, recruited from 2009 to 2017) [34,35], the Manitoba Tomorrow Project (n=~10,000, recruited from 2019 to 2024) [36], and Healthy Future Sask (n=~7000, recruited from 2022 to present) [37]. CanPath also includes CARTaGENE (n=43,000, recruited from 2009 to 2015) [38,39], which is participating in HEAL via study participants who have subsequently enrolled in NutriQuébec [25]. Broad eligibility criteria, encompassing adults aged 30 to 74 years who were able to complete surveys in English or French, were applied within the CanPath regional cohorts to achieve recruitment of a participant base that is generalizable [40], as almost half of adults in Canada fall into this age group [41].

CanPath is a rich resource of epidemiological data with multiple waves of follow-up data collection [40,42]. In addition to completing questionnaires on sociodemographic characteristics and a range of health-related measures, participants have provided physical measures and biological samples, including blood, urine, saliva, and toenails at baseline [40]. Consent has been obtained for passive follow-up via linkage to administrative health records and for inviting participants to complete additional measures within regional cohorts, along with coordinated efforts such as a cohort-wide COVID-19 questionnaire in 2021 [40,42]. Data are collected within each regional cohort and subsequently undergo a rigorous harmonization process [43].

#### NutriQuébec

In 2023 (prior to the funding of the HEAL study), CARTaGENE agreed to invite a subset of its participants to enroll in NutriQuébec. Thus, CARTaGENE is participating in HEAL via NutriQuébec, an ongoing open cohort of adults older than 18 years of age living in the province of Québec with a target enrollment of 20,000 participants and specific recruitment and retention strategies for low socioeconomic status populations [44]. NutriQuébec was launched in 2019 with the primary goal of providing essential data for the evaluation of the first-ever Government Health Prevention Policy in Québec [45]. The prevention policy was launched to influence factors that lead to better health status and quality of life and reduced social inequalities in Québec [45].

Participants are invited to complete yearly online questionnaires that query age, ethnicity, sex, gender identity, body weight, household composition, main occupation, education, annual income, global health status, household food security status, and 6-digit postal code for their place of residence [44]. NutriQuébec is collecting information on dietary intake and other measures using methodology similar to HEAL, described below [44].

#### Canadian Urban Environmental Health Research Consortium

The mission of CANUE is to advance knowledge on how characteristics of urban form, such as land use, physical infrastructure, and socioeconomic conditions, interact to affect health [46]. At its core is the collation and generation of standardized social and built environment data for every postal code in Canada [46]. CANUE is widely recognized as the premier source of urban environmental exposure data in Canada. CANUE’s established partnerships with CanPath and NutriQuébec provide the ability to access CANUE data linked to participants’ data through the established cohort data access procedures [47].

Geospatial exposures are captured with ArcGIS (Esri), which associates single-link DMTI Spatial postal codes to Statistics Canada dissemination area boundary files. Dissemination area identifiers will be used to join Census data with postal codes for participants within CanPath and NutriQuébec. Available variables include sprawl, population density, walkability, and social environment constructs such as gentrification, the Canadian Marginalization Index, and social and material deprivation [48].

#### Canadian Food Environment Dataset

Can-FED is the first high-quality geographic-based set of measures that represent spatial access to food in communities across Canada. Can-FED was developed through collaborations between researchers and Statistics Canada [27,49] and has been updated as part of HEAL. Can-FED measures are included in CANUE’s data platform, are linked to CanPath data, and are linkable to NutriQuébec. Can-FED uses existing microdata from the Business Register, an administrative dataset maintained by Statistics Canada, to develop measures of the RFE [49]. The resultant database provides standardized measures that can be applied to assess the RFE across jurisdictions in Canada at the dissemination area level.

Food outlets are extracted and classified from the Business Register based on an exhaustive list of 19 categories (eg, grocery stores and convenience stores) [49]. Food outlets are then mapped in a geographic information system using their addresses. RFE measures are calculated at the absolute (eg, counts per kilometer within a buffer of 1 and 3 km around the centroid of each dissemination area in Canada) and relative (eg, proportion of food outlets that sell a wide selection of fresh and nutritious food) levels [49]. Two relative density measures reflecting availability include the modified RFE index (the proportion of food outlets that sell a wide selection of fresh and nutritious food) and fast-food restaurant mix (the proportion of fast-food outlets relative to fast-food outlets and full-service restaurants) [49,50]. In total, 19 continuous measures, including densities of chain supermarkets, convenience stores, grocery stores, fast-food outlets, and fruit and vegetable markets, are calculated [49]. Validity of Can-FED has been examined in comparison with “gold standard” criteria, such as Enhanced Points of Interest data by DMTI Spatial Inc and data from a municipal health inspection list [49]. Geographic conversion tools, such as the Postal Code Conversion File [51], are used to link Can-FED to CanPath and NutriQuébec participants.

### Study Governance

The HEAL study included a 16-month planning period to facilitate coordination across the regional cohorts and engagement with external experts, knowledge users, and participants to refine the study design. The study design was informed by consultations with CanPath operational staff, a HEAL Working Group, a HEAL Scientific Advisory Committee, and participants. Decision-making rests with the Working Group, with oversight from the Scientific Advisory Committee.

The HEAL Working Group is comprised of the HEAL principal investigators, the scientific and operational leads from CanPath and the regional cohorts, the scientific lead of NutriQuébec, statistical experts, and representation from Maelstrom Research, which oversees data harmonization within CanPath and NutriQuébec. The Working Group is engaged approximately quarterly to discuss issues such as ethics applications, harmonizing the demographic data collected in HEAL with data already held by the regional cohorts, and specifications for the dietary intake and movement behavior assessments.

The Scientific Advisory Committee is comprised of experts in nutrition, dietary and movement behavior assessment, health policy and promotion, and biostatistics and epidemiology. The Committee includes knowledge users—including representatives from Health Canada and the Canadian Cancer Society—and CanPath, NutriQuébec, and Can-FED investigators. The Scientific Advisory Committee is engaged at least twice yearly to help ensure the relevance of the study, including the utility of the data collected beyond addressing the HEAL study objectives.

Alberta’s Tomorrow Project has a Participant Advisory Committee comprised of 30 participants who advise and inform decision-making on activities related to the design of ancillary studies, data collection tools, and communication materials. The Committee members are diverse with respect to sex, gender identity, age, geographic location in Alberta, and occupational backgrounds. The Participant Advisory Committee is engaged 2 to 3 times per year and has provided feedback on the HEAL study design, baseline questionnaire, and participant materials, including invitation letters and consent forms. The development of the participant materials was supported by CanPath’s national communications staff.

### Data Collection

#### Overview

Data collection tools and procedures are first described for the regional cohorts aside from CARTaGENE, followed by a description of the NutriQuébec study, that is, the source of dietary intake and movement behavior data for a subset of CARTaGENE participants.

**Figure 2:**
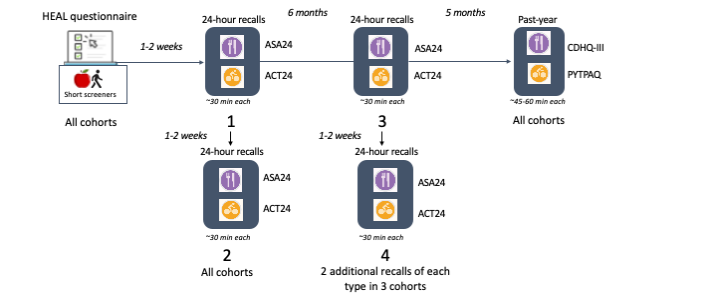
Collection of dietary intake and physical activity data within the HEAL study. ACT24: Activities Completed Over Time in 24 Hours; ASA24: Automated Self-Administered 24-Hour Dietary Assessment Tool; CDHQ-III: Canadian Diet History Questionnaire-III; HEAL: Healthy Eating and Supportive Environments; PYTPAQ: Past-Year Total Physical Activity Questionnaire.

CanPath regional cohorts hold email addresses for respective cohort participants. All regional cohort participants with an email and who can complete online questionnaires are eligible to participate in HEAL. Invitations to complete questionnaires and assessments are sent via email, and all assessments are completed online on desktop or laptop computers or on mobile devices, with responses entered manually by participants. All questionnaires use adaptive questioning, with some questions displayed based on the responses to other items. Participants are asked to complete a baseline questionnaire and a combination of tools to capture dietary intake and movement behavior over approximately a 1-year period per participant (Figure 2). To mitigate challenges with digital literacy, participants’ frequently asked questions and instruction guides for completing the questionnaires were adapted or developed. Call center staff at regional cohorts are available and are provided with frequently asked questions to assist participants. Data are collected regionally, integrated, and held by regional cohorts and the CanPath National Coordinating Centre [52].

#### HEAL Baseline Questionnaire

At baseline, participants in each regional cohort are invited to complete a web-based questionnaire. The questionnaire content (Table 1) was informed by the HEAL Working Group and Scientific Advisory Committee and reflects measures from different levels within the socioecological model [53] that are associated with dietary intake, movement behaviors, and chronic disease. Information collected includes gender identity, household income, financial stress, self-rated health, household food security status, and 6-digit postal code of residence and place of employment (if applicable). Participants are asked to provide their date of birth for verification purposes. The questions were drawn from prior CanPath questionnaires whenever possible to facilitate longitudinal analyses (eg, demographics, anthropometrics, physical and emotional health, and food security), along with established tools and measures used in other population cohort studies (eg, functional status and activities of daily living; Table 1).

**Table 1:** Content included in the Healthy Eating and Supportive Environments study baseline questionnaire.

Question category		Description	References
Demographics		Date of birth, sex, gender, pregnancy, breastfeeding, residential postal code, marital status, employment status, employment postal code, household income, household structure (number of children and adults living in household).	[54]
Financial stress		Feelings of stress due to finances.	[54,55]
Food security		Food situation in participants’ household in the prior year.	[56]
Experiences of discrimination		Experiences of discrimination as a result of sex, gender, ethnicity or culture, race or color, physical appearance, religion, sexual orientation, age, disability, language, or another reason.	[57]
Anthropometrics		Weight, height, waist, and hip measurement (self-measured).	[54]
Food timing		Timing of eating occasions over the prior year.	[58]
Diet quality (dietary screener)		Alignment of dietary intake to the 2019 Canada’s Food Guide healthy food choices recommendations over the past month.	[59,60]
Physical activity questionnaire		27 Questions on job-, transportation-, housework-, and recreational-related physical activity, as well as time spent sitting.	[61]
**Questions cohorts may choose to include**			
	Functional status	14-Item scale on upper body limitations, lower body limitations, and dexterity limitations.	[62]
	Physical and emotional health	The Patient Health Questionnaire, Generalized Anxiety Disorder, and self-reported health.	[63,64]
	Activities of daily living	20-Item scale capturing activities of daily living, for example, Can you dress and undress yourself without help?	[62]

#### Dietary Intake and Movement Behavior Assessment Tools

Participants who complete the baseline questionnaire are asked to complete various web-based dietary intake [65] and movement behavior assessments [66,67] over approximately a 1-year period (Table 2). The baseline questionnaire includes short dietary intake and movement behavior screeners, including the Canadian Food Intake Screener [59,60] and the long-form International Physical Activity Questionnaire, which queries movement behaviors over the past 7 days [61]. The Canadian Food Intake Screener [59,60], which provides a rapid assessment of dietary intake over the past 30 days in relation to the 2019 Canada’s Food Guide, is included to allow the regional cohorts to develop scoring algorithms based on comparison of the screener data to 24-hour dietary recall data [68]. Such scoring algorithms may be useful for improving the quality of dietary intake data from the screener for future data collections that do not include more intensive dietary assessment. Regional cohorts have previously used the International Physical Activity Questionnaire in baseline data collections, providing an opportunity for longitudinal analyses, while the Canadian Food Intake Screener has been administered in Alberta’s Tomorrow Project and will be newly administered in the other regional cohorts.

**Table 2:** Dietary intake and movement behavior assessment tools administered to participants within the Canadian Partnership for Tomorrow’s Health (CanPath) regional cohorts as part of the Healthy Eating and Supportive Environments (HEAL) study.

Assessment tool		Description	Administration considerations	Psychometric properties
**Dietary intake**				
	Canadian Food Intake Screener [59,60]	16-Item screener that rapidly assesses alignment of intake with the 2019 Canada’s Food Guide healthy food choices recommendations. Application of the scoring algorithm results in a total score that ranges from 0 to 65, with higher scores reflecting higher alignment with the guidance.	Requires ~5 minutes to complete. Available in English and French. Can be programmed into any survey platform.	A similar level of construct validity as observed for other multidimensional screeners has been shown in a sample of adults [60].
	Automated Self-Administered 24-Hour Dietary Assessment Tool (ASA24^a^-Canada-2018) [65,69]	Allows web-based self-administration of 24-hour dietary recalls, and auto-codes reported foods and beverages, eliminating the need for trained interviewers and coders. Includes multiple passes adapted from the United States Department of Agriculture’s Automated Multiple-Pass Method to enhance the accuracy and completeness of recall. Output includes all foods and beverages reported and estimates of energy and nutrient intake per food and per recall.	Requires 30 to 60 minutes to complete a recall. Available in English and French. Single sign-on can be used to integrate ASA24-Canada-2018 into an existing survey platform, eliminating the need for participants to sign in to a separate platform.	Feeding studies have shown that ASA24 captures intake with a high level of accuracy relative to true intake, ascertained through weighing of amounts served and plate waste, among samples of US adults [70].
	Canadian Diet History Questionnaire-III [71,72]	Past-year food frequency questionnaire that asks participants to recall intake of over 140 foods and beverages over the past year, including typical portion size and frequency of intake. Adapted from the US Diet History Questionnaire-III to reflect the Canadian food supply using data from the 2015 Canadian Community Health Survey—Nutrition.	Requires 45 to 60 minutes to complete. Available in English and French.	The US Diet History Questionnaire has been shown to have similar or better assessment of nutrients versus other food frequency questionnaires [73].
**Physical activity**				
	International Physical Activity Questionnaire-Long Form (IPAQ-LF) [61,74]	27-Item questionnaire that asks participants to report the frequency and duration of vigorous and moderate intensity physical activity, as well as walking, in the prior 7 days across occupational, transportation, household, and recreation or leisure domains.	Requires 10 to 15 minutes to complete. Available in English and French. Can be programmed into any survey platform.	Strong associations observed with data from an activity monitor and total and vigorous activity estimated by the IPAQ-LF among adults [74].
	Activities Completed Over Time in 24 Hours (ACT24) [66,75]	Allows web-based self-administration of 24-hour activity recalls capturing a range of sedentary behaviors, including screen time, as well as low-intensity physical activity, daily activities, and activity intensities over time. Output includes total energy expenditure, physical activity energy expenditure, physical activity level, physical activity time, sedentary time, and measures of sleep.	Requires 30 to 60 minutes to complete among individuals aged 50- to 74-years (the predominant age range in CanPath) [76]. Available in English and undergoing translation to French for the HEAL study. Personalized participant links can be used, eliminating the need for participants to sign in to a separate platform.	Good agreement with total energy expenditure based on doubly labeled water and activPAL accelerometer values [76]. Higher correlations with doubly labeled water-measured physical activity energy expenditure compared to other questionnaires [76]. Higher correlations with accelerometer (activPAL) active and sedentary time compared to other questionnaires [77]. Mean difference in sedentary time of 1.9% between ACT24 and accelerometry [77].
	Past-Year Total Physical Activity Questionnaire [67]	Past-year frequency questionnaire that captures type, duration, frequency, and intensity of physical activities in the previous 12 months, including employment and volunteer activities (including walking or biking to and from); household, childcare, and do-it yourself activities; and recreation and leisure activities. Output includes total hours per week spent in each activity; total physical activity quantified in metabolic equivalent hours per week; and frequency, duration, and perceived intensity of activities reported over the past year.	Requires 30 to 60 minutes to complete. Available in English and undergoing translation to French for the HEAL study.	Similar reliability and validity for measuring past-year physical activity relative to accelerometer and 7-day activity logs as other self-administered activity questionnaires [67].

^a^ASA24: Automated Self-Administered 24-Hour Dietary Assessment Tool.

A total of 1 to 2 weeks following completion of the baseline questionnaire, participants are invited via email to complete an online 24-hour dietary recall using the Automated Self-Administered 24-Hour Dietary Assessment Tool (ASA24-Canada-2018) [65,69] and a 24-hour physical activity recall using Activities Completed Over Time in 24 Hours (ACT24) [66,75]. Participants are invited to complete a second set of recalls 1 to 2 weeks later. A subset of cohorts, including Alberta’s Tomorrow Project, Manitoba’s Tomorrow Project, and Atlantic Partnership for Tomorrow’s Health, invites participants to complete another 2 sets of ASA24-Canada-2018 and ACT24 recalls 6 months later. The 24-hour dietary recalls aim to collect information on all foods, beverages, and supplements consumed the prior day from midnight to midnight. Similarly, the 24-hour activity recall queries all activities in the prior day, including time spent in bed, sedentary behavior, and physical activity. One year after baseline, participants are asked to complete past-year food and physical activity frequency questionnaires. These include the Canadian Diet History Questionnaire-III (CDHQ-III) [71,72] and the Past-Year Total Physical Activity Questionnaire (PYTPAQ) [67]. Prior versions of the Canadian Diet History Questionnaire have been administered in Alberta’s Tomorrow Project and CARTaGENE [38,72], and the PYTPAQ was included in the Alberta’s Tomorrow Project baseline questionnaires administered from 2000 to 2008 [29]. The ASA24 has been administered to subsets of participants in the BC Generations Project (unpublished) and Alberta’s Tomorrow Project [78], while the ACT24 is being newly administered to CanPath participants as part of HEAL.

The 24-hour recalls are the primary source of dietary intake data used to address the objectives of the HEAL study. The CDHQ-III is administered to broaden possible applications of the data in the future. Using data from dietary recalls and frequency questionnaires in epidemiological research leverages the strengths of each, increasing power and precision for estimation of associations with dietary intake [79-81]. Validation studies comparing self-report data to recovery biomarkers have shown that systematic error (ie, bias) in estimates of absolute intake is greater in food frequency questionnaires (FFQs) versus recall data [81-85], raising concerns about the use of FFQ data alone [86]. Comparatively, data from 24-hour recalls are affected to a larger extent by random error, largely due to within-person day-to-day variation [87,88], which can be addressed using repeat measures and statistical modeling [89,90]. Nonetheless, 24-hour recalls may not adequately capture infrequently consumed components, such as fish, whereas this is a strength of FFQs, given their focus on a longer period [91]. Overall, the 24-hour recall data will provide estimates of total intake of foods and nutrients, whereas the FFQ data will lend insights into less frequently consumed foods. The 2 tools can be used together in future research, for example, using regression calibration to mitigate bias in estimates of diet-disease associations [92].

### Data Collection Procedures

The data collection approach was informed by prior feasibility work in Alberta’s Tomorrow Project [78] and an expert advisory committee that provided guidance to Alberta’s Tomorrow Project in 2019. The regional cohort was planning a comprehensive diet and movement behavior assessment that was interrupted by the COVID-19 pandemic. Data collection will be staggered across cohorts (eg, one cohort starts in January, another in April to facilitate sharing of “lessons learned” and adjustment to implementation as needed). The study was announced to CanPath participants through existing CanPath and cohort communication channels, including websites, newsletters, and heads-up emails notifying participants of the study in advance of the invitation. Within each cohort, emails inviting participants to complete the baseline questionnaire are sent in waves, with the aim of spreading the subsequent 24-hour recalls over the months of the year to minimize the effects of seasonality. Phased rollout across cohorts across the months of the year further minimizes the effects of seasonality at the population level. Each participant completes their data collection activities over about a 1-year period, with full data collection for all participants in each regional cohort expected to last from 1.5 to 2 years.

Participants enter the study by completing the HEAL baseline questionnaire. Participants are able to return to prior questions to change their answers, but not to advance in the questionnaire without providing a response. Up to 2 email reminders to complete the questionnaire will be sent to participants who have not responded after receiving the initial email invitation. The reminders will be sent 14 and 21 days after the initial invitation.

Participants who complete at least the demographic section of the HEAL baseline questionnaire are invited within approximately 2 weeks to complete their first ASA24-Canada-2018 and ACT24 recalls. To reduce participant burden, personalized hyperlinks or single sign-on methods are used. These methods will enable participants to access the recall systems directly from their email invitation or their regional online participant platform without being required to enter unique usernames and passwords for ASA24-Canada-2018 and ACT24. Automated batching of output files from ASA24-Canada-2018 and ACT24 can be used by cohorts to facilitate tracking participants’ completion status and sending reminders.

Invitations to complete ASA24-Canada-2018 and ACT24 recalls are sent in the morning, prompting participants to report their intake and activity for the prior day from midnight to midnight. The unannounced recalls are intended to reduce reactivity [91]. Participants are encouraged to complete the recalls on the day they receive the invitation. The recalls can be completed at any time during the day (up to 8 AM the following day for ASA24-Canada-2018) and in multiple sittings if needed. Nonresponders receive up to 3 reminders every 3 days. Once the first set of recalls has been completed, participants are invited to complete another set of 2 recalls (1 diet and 1 activity, for a total of 2 of each type) within 3 to 10 days. Approximately 6 months later, participants in Alberta’s Tomorrow Project, Manitoba’s Tomorrow Project, and Atlantic Partnership for Tomorrow’s Health are asked to repeat the recall protocol for a total of 4 of each type (Figure 2). The additional recalls will contribute data to better estimate the usual diet and movement behavior.

Approximately 1 year after completing the baseline questionnaire, participants are invited via email to complete the CDHQ-III and PYTPAQ. Nonresponders receive up to 3 reminders 7, 14, and 25 days later.

The time to complete the baseline questionnaire and all diet and physical assessment tools is approximately 4 to 5 hours for participants who complete 2 ASA24-Canada-2018 and 2 ACT24 recalls and 6 to 7 hours for participants who complete an additional 2 ASA24-Canada-2018 and an additional 2 ACT24 recalls. The ASA24-Canada-2018, ACT24, and CDHQ-III systems all have researcher platforms that allow for monitoring of completion status. These platforms provide an indication of whether the recalls and questionnaires have been completed by each participant. Data cleaning will be conducted to consider whether recalls and questionnaires are sufficiently complete even if participants did not make it all the way to the end or alternatively, whether some recalls and questionnaires that appear to be complete do not represent high-quality data. For example, for the dietary recalls, established steps include checking for outliers in estimated nutrient intakes [93].

### NutriQuébec

NutriQuébec uses an online 24-hour dietary recall platform, Rappel de 24 h Web (R24W), that was developed for use in Québec [44,94,95]. Like ASA24 (Table 2), participants report foods and beverages consumed the previous day by browsing or using the search engine. Additionally, prompts query the context of consumption (time, place, and presence of others) and remind participants about potentially forgotten food categories. The R24W can be used on a phone, tablet, or computer. NutriQuébec participants receive unannounced email invitations to complete 2 to 3 recalls using R24W on nonconsecutive days over a 30-day period [96,97], with up to 5 reminders for nonresponders. The NutriQuébec study is collecting movement behavior data using the Physical Activity and Sedentary Behavior Questionnaire, developed by the PULSAR’s *Comité Activité physique et sédentarité* [98]. The questionnaire includes items from the Godin Leisure-Time Exercise Questionnaire [99], the European Prospective Investigation into Cancer and Nutrition physical activity questionnaire [100], and the Québec Population Health Survey [101]. A recent study among 48 female and 20 male population in Québec found that data generated from 2 R24W and 2 ASA24-Canada dietary recalls were comparable, with no meaningful differences in estimated intake of energy, proteins, lipids, saturated fatty acids, carbohydrates, fibers, sodium, and vegetables and fruits [94].

### Data Integration and Harmonization

Data from the HEAL baseline questionnaire and dietary intake and movement behavior assessment tools will be transferred to Maelstrom Research [102] for cleaning and integration with existing CanPath and NutriQuébec data. Each participant has a unique study ID, which facilitates data integration with earlier waves of data collection within each respective regional cohort. Existing CanPath data are already connected to Maelstrom’s platform. Cleaning procedures include those specifically developed for 24-hour dietary and movement behavior recalls, which encompass examination and assessment of incomplete recalls, as noted previously [43,93]. The 6-digit postal code of participants’ residence and work (if applicable) allows linkage to geospatial environment data from CANUE and Can-FED [67]. Collection of data using the same tools and data dictionaries across cohorts (aside from NutriQuébec) minimizes the need for harmonization. Harmonization of data between CanPath and NutriQuébec will follow best practices outlined by Fortier et al [43] including defining target variables and harmonization potential, processing of data, and estimating the quality of the generated harmonized dataset.

### Data Access

All data generated via HEAL will be available within the CanPath researcher access platform using existing access request procedures detailed on the CanPath website.

### Statistical Analysis

The association between a given food environment characteristic (ie, density of fast food outlets) with diet quality will be evaluated using generalized linear mixed models, both unadjusted and adjusted for possible confounders as fixed effects. A random intercept representing the dissemination area (unit of measurement of the food environment) will be included. The mixed component of the generalized linear mixed model will contain random effects to account for the correlation of participants within dissemination areas. Unexplained variation will be partitioned into between- and within-area components and between-area heterogeneity in the effects of environmental exposure variables. Effect modification will be used to determine whether associations between the food environment and diet quality vary by environmental- and individual-level chronic disease risk factors.

### Ethical Considerations

Ethics approval for HEAL was granted by the University of British Columbia/BC Cancer Institutional Review Board (H23-02478). Each CanPath regional cohort study will also obtain local ethics approval for its respective data collection. Participants in CanPath provide consent to be recontacted for research purposes and for secondary analysis of data in the future. Participants are recontacted and asked to provide informed consent if they wish to participate in HEAL. The consent process included details on the purpose of HEAL, study investigators, the length of each survey, and data storage and retention. The regional cohorts retain personal identifiers. For verification purposes, participants are asked to provide their birthdate for questionnaires completed, but the HEAL research team will not receive this information. Only deidentified data will be accessible to the HEAL research team and to investigators who receive approval for use of the data. Participants do not receive compensation for participation.

## Results

Participant recruitment and data collection began in spring 2025 in 2 regional cohorts: Manitoba’s Tomorrow Project and the BC Generations Project. Participant recruitment in the remaining cohorts will occur until fall 2026. Data collection is expected to conclude by the end of 2027. As of manuscript acceptance (November 4, 2025), 3171 participants had been recruited. Data cleaning and harmonization will occur on a rolling basis. Linkage of data collected within HEAL to CANUE and Can-FED data occurs twice per year to coincide with the transfer of cleaned or harmonized data from Maelstrom to the regional cohorts. Analysis of the main study aims pertaining to dietary intake and the food environment is planned for 2026. It is anticipated that dietary intake and movement behavior data will be available for up to 100,000 participants and accessible to researchers in 2029. At the end of the study, participants will receive a report summarizing the diet and movement behavior information they provided.

## Discussion

### Principal Findings

This protocol outlines the extensive planning and coordination required for the collection of detailed dietary and movement behavior data that will be linked to rich environmental data, along with the wealth of information already held by regional cohorts in Canada. The approach of embedding data collection within ongoing cohorts is resource sparing, drawing on the significant investments already made in the CanPath and NutriQuébec infrastructure. In addition to an existing pool of participants, CanPath is a research platform, meaning that data collected by the regional cohorts are available for use by the broader research community in Canada and internationally. This protocol may inform the design and collection of detailed dietary and movement behavior assessments using web-based tools in other epidemiologic and surveillance studies.

The comprehensive data collection, including sociodemographic data and completion of multiple dietary and movement behavior tools, will enable us to address the aims of HEAL, improving our understanding of how environmental factors are associated with dietary intake among adults in Canada. The HEAL study thus shifts focus from individual-level behaviors and choices to the broader structural environment, recognizing the complexity of factors that shape dietary intake. The new dietary and movement behavior data collected within HEAL have implications more broadly as CanPath is designed to facilitate collaboration with large-scale international cohorts, including the UK Biobank [103] and the European Prospective Investigation into Cancer and Nutrition study [104], both of which hold detailed dietary and movement behavior data.

### Strengths and Limitations

This work will provide a platform for addressing a wealth of research questions as the largest detailed assessment of dietary intake and movement behavior in Canada. The study population, which spans all 10 provinces of Canada and includes structurally excluded groups, is a major strength. The large sample will allow consideration of how factors such as gender identity and income interact with environmental factors in relation to diet. The combination of assessment tools will support methodological research, including expanding strategies, to mitigate the effects of error in dietary intake and movement behavior data on observed diet-disease associations. The collection of data using multiple tools in a large population is uniquely possible because of the advent of web-based systems that enhance the feasibility of recalls by eliminating the need for interviewers and coders and that allow for automated batching of output to facilitate participant tracking and reminders.

A limitation is the further introduction of self-selection in this volunteer sample of participants through the baseline questionnaire, which serves as a gateway to the HEAL study. Given the time commitment and collection of data over a 1-year period, participant nonresponse is also anticipated. However, a prior substudy in Alberta’s Tomorrow Project, in which participants were asked to complete 4 ASA24 recalls and an online frequency questionnaire within 4 months, suggests that our approach will have reasonable uptake. Of participants who consented to the Alberta Tomorrow Project substudy (~50% of those invited), 70% completed at least 1 tool and half completed more than 1 recall [78].

The lack of comprehensive data in Canada is a barrier to training emerging researchers in the areas of diet and movement behavior assessment and epidemiology. The enrichment of CanPath through HEAL provides a valuable opportunity to expand capacity in these areas in Canada, which is crucial given that diet and movement behavior patterns are not aligned with guidance [1,105], with substantial implications for morbidity and mortality [9,106].

### Conclusions

In the immediate term, the data newly collected in HEAL will lend insights into how features of the built, social, and RFE are associated with dietary intake in a large population of adults in Canada. In the longer-term, data collected in HEAL will provide an unprecedented opportunity to advance research on nutrition and movement behavior. For instance, data from the recalls and past-year questionnaires can be leveraged to examine associations with long-term outcomes through linkage to administrative health data including vital statistics, hospital records, and cancer registry data. This effort will also build capacity in the collection, processing, and harmonization of dietary intake data among Canadian cohorts and provide a training ground for emerging researchers.

## Data Availability

Data generated through this protocol will be available upon approval from the data access committee of the Canadian Partnership for Tomorrow’s Health.

## Appendix

Multimedia Appendix 1 Peer review report by Nutrition, Food & Health/Nutrition, aliments et santé Committee, Canadian Institutes of Health Research (CIHR).

## Abbreviations

ACT24: Activities Completed Over Time in 24 Hours
ASA24: Automated Self-Administered 24-Hour Dietary Assessment Tool
Can-FED: Canadian Food Environment Dataset
CanPath: Canadian Partnership for Tomorrow’s Health
CANUE: Canadian Urban Environmental Health Research Consortium
CDHQ-III: Canadian Diet History Questionnaire-III
FFQ: food frequency questionnaire
HEAL: Healthy Eating and Supportive Environments
PYTPAQ: Past-Year Total Physical Activity Questionnaire
RFE: retail food environment
R24W: Rappel de 24 h Web
