# Supplementary material for: Enrichment of the Canadian Partnership for Tomorrow’s Health Study: Protocol for Administering Multiple Online Dietary and Movement Behavior Assessment Tools in a Longitudinal Cohort Study
Source: JMIR Res Protoc. 2025 Dec 2;14:e71680. doi: 10.2196/71680 (PMC12709159; doi:10.2196/71680)
Supplement: Multimedia Appendix 1 [file resprot_v14i1e71680_app1.pdf]

**Review Type / Type d'évaluation:**	Reviewer 1 / Évaluateur 1
**Name of Applicant / Nom du chercheur:**	Murphy, Rachel
**Application No. / Numéro de demande:**	496120
**Agency / Agence:**	CIHR/IRSC
**Competition / Concours:**	Project Grant/Subvention Projet
**Committee / Comité:**	Nutrition, Food & Health/Nutrition, aliments et santé
**Title / Titre:**	HEALthy Eating and Supportive Environments (HEAL); a Pan-Canadian study

Adjudication Criteria/Critères de sélection

Initial Score/Cote Initiale: 4.0

Top/Bottom Selection/Groupe supérieur/inférieur

- (●) **Top/Groupe supérieur**
- ( ) **Bottom/Groupe inférieur**

**Review Type / Type d'évaluation:**	Reviewer 1 / Évaluateur 1
**Name of Applicant / Nom du chercheur:**	Murphy, Rachel
**Application No. / Numéro de demande:**	496120
**Agency / Agence:**	CIHR/IRSC
**Competition / Concours:**	Project Grant/Subvention Projet
**Committee / Comité:**	Nutrition, Food & Health/Nutrition, aliments et santé
**Title / Titre:**	HEALthy Eating and Supportive Environments (HEAL); a Pan-Canadian study

Summary of Application/Résumé de la demande:

The proposed study seeks to examine for the first time in Canada the relationship between retail food environment and diet quality, while taking into consideration neighbourhood (built and social) environment factors as well as individual-level covariates (sex, income, etc.). Specific goals include:

1) Evaluate associations between the retail food environment and diet quality in terms of alignment with the CFG-2019, at the community, provincial and national level in Canada.
2) Examine whether associations between the retail food environment and diet quality vary by built and social environment factors and by individual-level chronic disease risk factors.

These goals will be accomplished by capitalizing on a Pan-Canadian infrastructure of four linked national resources (the Canadian Partnership for Tomorrow's Health (CanPath), the NutriQuébec study, the Canadian Urban Environmental Health Research Consortium (CANUE), and the Canadian Food Environment Dataset (Can-FED)). Built and social environment factors such as urban sprawl, population density, walkability, material and social deprivation will be obtained through CANUE, which has already been linked to CanPath. Retail food environment estimates will be obtained through Can-FED, which is the first, high quality, geographic-based dataset of measures that represent spatial access to foods in communities across Canada. Measures in Can-FED (absolute and relative RFE) will need to be updated as part of the proposed work. The bulk of the effort will concentrate on gathering 24-hour dietary recall data from a large number of participants that are enrolled in CanPath. Two recalls are proposed using the ASA24 tool and alignment with the CFG-2019 will be measured by the Healthy Eating Food Index (HEFI)-2019.

In addition to diet quality, secondary outcomes will include nutrients (sodium, free sugars, saturated fats, unsaturated/saturated fats ratio), foods (protein foods, plant-based protein foods, vegetables and fruits, whole-grain foods, ratio of whole grains/all grain foods), and beverages (water relative to all beverages).

Strengths and Weaknesses/Forces et faiblesses:

Strengths:

I was keen to read this innovative and very topical proposal from a stellar research team with a wealth of expertise in leading large-scale investigations. The proposal is based on solid evidence and a strong rationale that improvements in dietary behaviours among individuals need to be supported by system-level environmental changes.

The use of existing infrastructure of the CanPath project synthesized with NutriQuebec, with data already linked to environmental characteristics available in CANUE and Can-FED is a clear strength. The data collection procedures are well thought out, well articulated, with a lot of key technical detail provided (page 5). The use of well-established tools and validated measures, including the self-administered ASA24-Canada based on automated multiple pass method and the Healthy Eating Food Index (HEFI-2019), is also a strength.

The evidence generated has the potential to inform system level changes to promote the creation of supportive environments to facilitate healthy eating. Strong letters of support from Health Canada and other key stakeholders are provided.

I appreciated the use of figures to help understand the complexity of the proposed data collection and timelines.

Weaknesses:

I understand that the retail food environment data available through Can-FED may be outdated since it captures the status of retail outlets in 2018 prior to COVID-19. While the ‘processed for accessing and using the Business Registry data, and the methodology for developing the RFE measure are already in place’ (page 7), the complexity and the time required to update this information is not clear (not articulated in the timeline). In addition, the CN-Marg and the Pampalon index of social and material deprivation are also dated (2016) with no date for the 2021 update available yet (the Appendix Table 4 indicates that both these indices are available in CANUE for 2021 but this is incorrect).

I am confused about the number of participants that will be surveyed as part of the proposed research to provide new dietary and physical activity data – the Summary says about 950,000 participants in CanPath plus those in the NutriQuebec will be surveyed. On page 4, the conservative estimate of about 950,000 is mentioned again, however in the same paragraph it is emphasized that the total number of participants will be about 136,000 and this is the number also included in the Figure on page 3.

My biggest concern however has to do with the feasibility of re-contacting participants. Pages 3-4 state that CanPath is not planning new waves of data collection at this time and that CanPath data are now more than 10 years old. It would be helpful to know when was the last contact with participants in the 7 existing cohorts. The CanPath is essentially being utilized or re-purposed as a sampling frame for this new study and it is not clear whether the feasibility of re-engaging these participants has been established. In addition, it would be helpful to articulate if and how any of the existing data already collected through CanPath will be utilized.

Further to this, the data collection for the proposed study shifts the mode of administration from paper to online, which requires email addresses of a large number of participants – previous data collections were administered by mail. It is not clear whether the investigators have in their possession the email addresses of all of these participants.

It is not clear why the Ontario Health Study, which is the largest and most diverse cohort, not included in this proposal and whether the objectives of understanding the health inequalities, particularly racial/ethnic inequalities) as articulated on page 9 can still be met without the inclusion of Ontario cohort in this effort.

The outsourcing of data cleaning and management to a commercial company hinders the oversight and involvement in decision-making by the PI and the research team.

Page 9 mentioned the nested data structure under limitations – it is not clear how these data are considered nested, more explanation is needed to understand this point.

**Review Type / Type d'évaluation:**	Reviewer 1 / Évaluateur 1
**Name of Applicant / Nom du chercheur:**	Murphy, Rachel
**Application No. / Numéro de demande:**	496120
**Agency / Agence:**	CIHR/IRSC
**Competition / Concours:**	Project Grant/Subvention Projet
**Committee / Comité:**	Nutrition, Food & Health/Nutrition, aliments et santé
**Title / Titre:**	HEALthy Eating and Supportive Environments (HEAL); a Pan-Canadian study

Budget Recommendation/Recommandation budgétaire:

No concerns.

Please indicate your appraisal of the integration of sex as a biological variable as a strength, weakness, or not applicable to the proposal./Prière de sélectionner une option pour donner votre évaluation de l'intégration du sexe comme variable biologique en tant que point fort ou point faible de la proposition, ou en tant qu'élément non applicable à la proposition.

- (●) **Strength/Point fort**
- ( ) **Weakness/Point faible**
- ( ) **Not applicable/Non applicable**

Please indicate your appraisal of the integration of gender as a socio-cultural determinant of health as a strength, weakness, or not applicable to the proposal./Prière de sélectionner une option pour donner votre évaluation de l'intégration du genre comme déterminant socioculturel de la santé en tant que point fort ou point faible de la proposition, ou en tant qu'élément non applicable à la proposition.

- (●) **Strength/Point fort**
- ( ) **Weakness/Point faible**
- ( ) **Not applicable/Non applicable**

**Review Type / Type d'évaluation:**	Reviewer 1 / Évaluateur 1
**Name of Applicant / Nom du chercheur:**	Murphy, Rachel
**Application No. / Numéro de demande:**	496120
**Agency / Agence:**	CIHR/IRSC
**Competition / Concours:**	Project Grant/Subvention Projet
**Committee / Comité:**	Nutrition, Food & Health/Nutrition, aliments et santé
**Title / Titre:**	HEALthy Eating and Supportive Environments (HEAL); a Pan-Canadian study

Sex and/or Gender Considerations/Notions de sexe et/ou de genre:

The consideration of gender and sex is discussed appropriately.

**Review Type / Type d'évaluation:**	Reviewer 2 / Évaluateur 2
**Name of Applicant / Nom du chercheur:**	Murphy, Rachel
**Application No. / Numéro de demande:**	496120
**Agency / Agence:**	CIHR/IRSC
**Competition / Concours:**	Project Grant/Subvention Projet
**Committee / Comité:**	Nutrition, Food & Health/Nutrition, aliments et santé
**Title / Titre:**	HEALthy Eating and Supportive Environments (HEAL); a Pan-Canadian study

Adjudication Criteria/Critères de sélection

Initial Score/Cote Initiale: 4.3

Top/Bottom Selection/Groupe supérieur/inférieur

- (●) **Top/Groupe supérieur**
- ( ) **Bottom/Groupe inférieur**

**Review Type / Type d'évaluation:**	Reviewer 2 / Évaluateur 2
**Name of Applicant / Nom du chercheur:**	Murphy, Rachel
**Application No. / Numéro de demande:**	496120
**Agency / Agence:**	CIHR/IRSC
**Competition / Concours:**	Project Grant/Subvention Projet
**Committee / Comité:**	Nutrition, Food & Health/Nutrition, aliments et santé
**Title / Titre:**	HEALthy Eating and Supportive Environments (HEAL); a Pan-Canadian study

Summary of Application/Résumé de la demande:

To drive meaningful, population-level changes in dietary intake across Canada, this group contends that large-scale robust studies are needed to identify food and neighbourhood environmental factors that shape dietary intake. The focus is to move beyond dietary intake as an individual behaviour, and examine and provide guidance about how the food environment can be shaped to support the behaviours that encourage healthy intake.

There are 2 main aims for this work.

Aim 1) Evaluate associations between the retail food environment (RFE) and diet quality (primary outcome is alignment with the CFG-2019) at the community, provincial and national level in Canada.
Aim 2) Examine whether associations between the RFE and diet quality vary by
a) built and social environment factors and
b) individual-level risk factors for chronic disease
Methods: The group will work with four existing cohorts that are part of CanPath, NutriQuébec, CANUE, and CanFED. Together this included individuals from 8 provinces, and leverages existing cohorts who have already collected extensive dietary data as well as important health data. They will then "layer" information from CANUE and Can-FED to understand the RFE, and explore the 2 main aims.

The plan is to administer the ASA24 – Canada (extensively tested and known to be one of the best tools available) and the Activities Completed Over Time in 24 Hours (ACT24 – captures sedentary behaviours including screen time as well as low-intensity physical activity, daily activities, and activity intensities over time) questionnaires to CanPath and the R24W to NutriQuebec participants.

They will combine all of this information with the CANUE information and use the combined dataset to explore the impacts of the food environment on dietary intake. CANUE's mission is to advance knowledge on how characteristics such as land use, physical infrastructure, and socioeconomic conditions, interact to affect health. CANUE has collated/generated standardized social and built environment data for every postal code in Canada. This group plans to focus on a few key aspects of this data, specifically sprawl, population density, walkability, and some social environment constructs (e.g. gentrification, the Canadian marginalization index (CAN-Marg), and two domains of deprivation (social and material deprivation) in exploring how they impact dietary intake. There is now a new part of CANUE (called Can-FED) that uses microdata from a Business Register whereby food outlets can be identified and characterized. Food outlets are then mapped by their location. This is another "layer" of information that will be considered.

**Review Type / Type d'évaluation:**	Reviewer 2 / Évaluateur 2
**Name of Applicant / Nom du chercheur:**	Murphy, Rachel
**Application No. / Numéro de demande:**	496120
**Agency / Agence:**	CIHR/IRSC
**Competition / Concours:**	Project Grant/Subvention Projet
**Committee / Comité:**	Nutrition, Food & Health/Nutrition, aliments et santé
**Title / Titre:**	HEALthy Eating and Supportive Environments (HEAL); a Pan-Canadian study

Strengths and Weaknesses/Forces et faiblesses:

Strengths:

•Outstanding team lead by a strong ECR with expertise examining dietary intake in relation to cancer prevention and control, and a wide range of factors that shape relationships between diet and disease prevention

•Ambitious project that brings together dietary intake and considers broad aspects of the food environment on dietary intake in a large-scale project with very good national representation.

•Leverages infrastructure of existing population health cohorts – CanPath + NutriQuebec.

•Cohort participants are "anticipating" additional follow up from the regional studies and therefore likely to have high response rates to request for additional information about dietary intake. NutriQuebec will leverage ongoing data collection protcols.

•Regional cohorts have experience coordinating and managing high volumes of online questionnaire administration.

•The combination of data that is brought together will help identify how the food environment shapes dietary intake in different parts of the country.

•There is a significantly shorter waiting time to disease outcomes than if a new cohort were started.

•Will contribute important updates to the Can-FED data post pandemic; the changes to the food environment have been extensive and this information can be used by others in the future.

•The primary outcome is diet quality, which will be assessed using the HEFI-2019. HEFI-2019 was developed (with extensive input/direction from several of the team members) to measure alignment of eating patterns to the CFG-2019, considering 10 components: vegetables and fruit, whole-grain foods, grain foods ratio, protein foods, plant-based protein foods, beverages, fatty acids ratio, saturated fats, free sugars and sodium.

•Focuses on the factors that shape individual dietary intake behaviours and looks beyond the factors at the individual. It is an exciting step in Canada to consider the complex array of factors that shape diet, across settings, sectors and societal levels. The possibility of regional/provincial comparisons is important.

•There is the potential to better appreciate how key aspects of the environmental setting may support healthy food choices. This project can examine impacts of community gardens, public markets, new/different grocery stores, etc. This information could inform community-level interventions that maximize the impact on healthy eating in Canada.

•There is potential to build research and implementation capacity in dietary and PA assessment as well as in community activism/structuring, planning, land use, policy development.

Weaknesses:

•The scope of this project is large and there are many possible snags that could emerge along the way. Would have been good to see some discussion of possible pitfalls and mitigation strategies.

•Is there any way that the investigators can learn from past waves pf data collection in these cohorts? E.g. examining dietary changes over time?

•What is the variability of social/demographic characteristics among these cohorts and how will this variation be dealt with in these analyses?

•How wide a “net” will be cast when defining the “local” food environment and its impact on dietary behaviours? People may not shop close to where they live or eat in local retail establishments. Has there been any consideration to how the wide array of delivery options that are available impacts purchasing and receiving foods? Does CANUE capture initiatives such as community-sponsored agriculture etc?

•Many of CanPath participants will be older adults? - will there be help for completing ASA24 and ACT24?

•KT strategies could be expanded to include broader range of local, provincial and national stakeholders.

**Review Type / Type d'évaluation:**	Reviewer 2 / Évaluateur 2
**Name of Applicant / Nom du chercheur:**	Murphy, Rachel
**Application No. / Numéro de demande:**	496120
**Agency / Agence:**	CIHR/IRSC
**Competition / Concours:**	Project Grant/Subvention Projet
**Committee / Comité:**	Nutrition, Food & Health/Nutrition, aliments et santé
**Title / Titre:**	HEALthy Eating and Supportive Environments (HEAL); a Pan-Canadian study

Budget Recommendation/Recommandation budgétaire:

no changes to the budget - accepted as described

Please indicate your appraisal of the integration of sex as a biological variable as a strength, weakness, or not applicable to the proposal./Prière de sélectionner une option pour donner votre évaluation de l'intégration du sexe comme variable biologique en tant que point fort ou point faible de la proposition, ou en tant qu'élément non applicable à la proposition.

- ◉ **Strength/Point fort**
- ◯ **Weakness/Point faible**
- ◯ **Not applicable/Non applicable**

Please indicate your appraisal of the integration of gender as a socio-cultural determinant of health as a strength, weakness, or not applicable to the proposal./Prière de sélectionner une option pour donner votre évaluation de l'intégration du genre comme déterminant socioculturel de la santé en tant que point fort ou point faible de la proposition, ou en tant qu'élément non applicable à la proposition.

- ◉ **Strength/Point fort**
- ◯ **Weakness/Point faible**
- ◯ **Not applicable/Non applicable**

**Review Type / Type d'évaluation:**	Reviewer 2 / Évaluateur 2
**Name of Applicant / Nom du chercheur:**	Murphy, Rachel
**Application No. / Numéro de demande:**	496120
**Agency / Agence:**	CIHR/IRSC
**Competition / Concours:**	Project Grant/Subvention Projet
**Committee / Comité:**	Nutrition, Food & Health/Nutrition, aliments et santé
**Title / Titre:**	HEALthy Eating and Supportive Environments (HEAL); a Pan-Canadian study

Sex and/or Gender Considerations/Notions de sexe et/ou de genre:

Described in appropriate amount of detail regarding: 1) asking about gender identity, 2) conducting analyses (in Aim2) using sex, gender and gender identify dimensions (gender roles and institutionalized gender) to examine differences by sex and gender-based differences. The study is powered (>80%) to detect meaningful differences among 400 participants (anticipated number expected to identify as a cultural gender minority)

**Review Type / Type d'évaluation:**	Reviewer 3 / Évaluateur 3
**Name of Applicant / Nom du chercheur:**	Murphy, Rachel
**Application No. / Numéro de demande:**	496120
**Agency / Agence:**	CIHR/IRSC
**Competition / Concours:**	Project Grant/Subvention Projet
**Committee / Comité:**	Nutrition, Food & Health/Nutrition, aliments et santé
**Title / Titre:**	HEALthy Eating and Supportive Environments (HEAL); a Pan-Canadian study

Adjudication Criteria/Critères de sélection

Initial Score/Cote Initiale: 4.4

Top/Bottom Selection/Groupe supérieur/inférieur

- (●) **Top/Groupe supérieur**
- ( ) **Bottom/Groupe inférieur**

**Review Type / Type d'évaluation:**	Reviewer 3 / Évaluateur 3
**Name of Applicant / Nom du chercheur:**	Murphy, Rachel
**Application No. / Numéro de demande:**	496120
**Agency / Agence:**	CIHR/IRSC
**Competition / Concours:**	Project Grant/Subvention Projet
**Committee / Comité:**	Nutrition, Food & Health/Nutrition, aliments et santé
**Title / Titre:**	HEALthy Eating and Supportive Environments (HEAL); a Pan-Canadian study

Summary of Application/Résumé de la demande:

Goal of the proposed project is to identify, for the first time in Canada, factors associated with the retail food environment (RFE) that shape dietary intake at the population level by leveraging four national resources of ongoing studies.

Methods: The plan is to leverage data available and access to participants for future survey in four, linked national resources, the Canadian Partnership for Tomorrow's Health (CanPath), the NutriQuébec study, the Canadian Urban Environmental Health Research Consortium (CANUE), and the Canadian Food Environment Dataset (Can-FED). Comprehensive dietary data will be collected in ~950,000 participants in CanPath using two Automated Self Administered 24-hour Dietary Assessments (ASA24-Canada). Data will be harmonized with dietary recall data collected on a yearly basis in the NutriQuébec study that is actively recruiting 20,000 adults 18y and older. Dietary data from CanPath and NutriQuébec will be integrated with CANUE, facilitating use of existing metrics on characteristics of urban form such as urban sprawl, walkability, material and social deprivation.

The study will utilize novel tools to collect high quality comprehensive dietary intake data, including multiple 24-hour recalls, paired with physical activity data, new (i.e. food security) and updated data on demographics and chronic disease risk factors. Despite the challenges in having to patch together existing/ongoing provincial and national initiatives, this study holds promise of an important first step in bringing a systems-science approach to developing Canadian dietary guidelines.

**Review Type / Type d'évaluation:**	Reviewer 3 / Évaluateur 3
**Name of Applicant / Nom du chercheur:**	Murphy, Rachel
**Application No. / Numéro de demande:**	496120
**Agency / Agence:**	CIHR/IRSC
**Competition / Concours:**	Project Grant/Subvention Projet
**Committee / Comité:**	Nutrition, Food & Health/Nutrition, aliments et santé
**Title / Titre:**	HEALthy Eating and Supportive Environments (HEAL); a Pan-Canadian study

Strengths and Weaknesses/Forces et faiblesses:

Significance: This ambitious project is highly relevant to advancing approaches to establishing dietary guidelines to improve the health of all Canadians irrespective of demography and SES. The use of systems-science approaches (with a focus on the food environment) and methods in the decision making for dietary guidelines (DG) was deemed essential to improve the rigor, integrity and trustworthiness of future DGs for Americans guidelines in a recent report From the National Academies titled: Evaluating the Process to Develop the DG for Americans (doi.org/10.17226/26653).

Feasibility:

•The plan to leverage four, linked national resources, the Canadian Partnership for Tomorrow’s Health (CanPath), the NutriQuébec study, the Canadian Urban Environmental Health Research Consortium (CANUE), and the Canadian Food Environment Dataset (Can-FED) could be challenging (Figure 1 was very helpful in visualizing the component parts of the population bases). However, the applicants are already associated with most of these projects. More importantly, they have engaged the collaboration of Maelstrom at McGill university (letter of support from Dr. Fortier) who have an outstanding reputation in harmonization of data across independent studies. Thus, I have great confidence in the success of merging the data for dietary and environmental factors across studies.

•Letters of support from leaders of the individual studies in addition to Canadian Cancer Society, Health Canada food Directorate and BC Cancer are very enthusiastic and supportive which is important for knowledge mobilization.

Expertise

•The applicants include ECRs, mid- and senior career researchers. Drs. Murphy is well trained and highly accomplished at an early career stage in the fields of nutrition epidemiology 12/33 peer reviewed papers in 2021-22 as first or senior author despit LOA in 20-21 and Covid consequences to work productivity. Dr. Kirpatrick’s expertise lies in dietary assessment in which her research program aims to improve methodologies for measuring dietary patterns to generate evidence to inform healthy eating. She was a member of the National Academies expert committee on DGAs noted above so is very attuned to the importance of systems-science in developing nutrition guidelines.

Weaknesses

- Systems-science is a new area in nutrition so some background and specific examples of the applicability of the data to be generated to development of dietary guidelines would be helpful.
- No hypothesis
- KT strategies are described very generally.
- There is no mention of seasonal variation in food availability within a location or between locations. It appears that the diet records were not targeted to a specific season.

Budget Recommendation/Recommandation budgétaire:

Budget – request for $2 265 000 over 5 years

Personnel to support data collection at all sites is $1,659,957, which is difficult to evaluate.

Trainees – 3 PhD seem reasonable. Not sure that providing each with a laptop computer is necessary - total ~$7,000.

KT – includes 2 trainee workshops to facilitate networking and collaboration opportunities, and allow cross-pollination across sub-analyses, $6,500 x 2 = $13,000 but details are not provided such as whether it is for just the 3 trainees on the project or for the exposure of the project and education of other trainees. Such gatherings could be accommodated at national meetings. Travel for the Steering Committee could be eliminated and replaced by virtual or hybrid meetings which have proven to be effective during the pandemic.

Please indicate your appraisal of the integration of sex as a biological variable as a strength, weakness, or not applicable to the proposal./Prière de sélectionner une option pour donner votre évaluation de l'intégration du sexe comme variable biologique en tant que point fort ou point faible de la proposition, ou en tant qu'élément non applicable à la proposition.

- ◉ **Strength/Point fort**
- ◯ **Weakness/Point faible**
- ◯ **Not applicable/Non applicable**

Please indicate your appraisal of the integration of gender as a socio-cultural determinant of health as a strength, weakness, or not applicable to the proposal./Prière de sélectionner une option pour donner votre évaluation de l'intégration du genre comme déterminant socioculturel de la santé en tant que point fort ou point faible de la proposition, ou en tant qu'élément non applicable à la proposition.

- ◉ **Strength/Point fort**
- ◯ **Weakness/Point faible**
- ◯ **Not applicable/Non applicable**

Sex and/or Gender Considerations/Notions de sexe et/ou de genre:

Gender is addressed in relation to how data has been collected across surveys included in the project so they have an idea of gender identity prevalence across studies. Since gender identity is fluid the applicants have included a relevant question in the data to be collected.

For aim 2, effect modification by sex, gender, and gender dimensions (focused on gender roles and institutionalised gender such as family structure, occupation segregation and level of education) will be assessed to determine potential sex and gender-based differences and power analysis is provided to detect a meaningful difference in diet quality per one unit difference in retail food environment measure among 400 participants as >80%.
